# Lebanese women׳s awareness and attitude toward epidural anesthesia during labor

**DOI:** 10.1016/j.dib.2018.05.051

**Published:** 2018-05-23

**Authors:** M. Fawaz, S. Malas

**Affiliations:** a Beirut Arab University, Faculty of Health Sciences, Lebanon; b Beirut Arab University, Faculty of Medicine, Department of Obstetrics and Gynecology, Lebanon

**Keywords:** Awareness, Attitude, Knowledge, EA, Labor pain

## Abstract

Women׳s choice to use epidural anesthesia (EA) for relief of labor pain varies from one culture to another. A descriptive cross-sectional design was used to gather data from a sample of 200 women in childbearing age. Data was gathered from general population in Lebanon. Demographic data, knowledge and attitudes questionnaires towards EA were used to gather data for this study. The data in this article provides demographic data about Lebanese women and their awareness and attitudes towards epidural anesthesia. The analyzed data is provided in the tables included in this article.

**Specifications Table**

**Table:** 

Subject area	*Nursing*
More specific subject area	*Obstetric and Gynecological Nursing*
Type of data	*Tables*
How data was acquired	*Quantitative*
Data format	*Analyzed*
Experimental factors	*No experimental factors*
Experimental features	*No experimental features*
Data source location	*Lebanon*
Data accessibility	*Data is available within this article*
Related research article	*Mohamed, H. F., Alqahtani, J., Almobaya, N., Aldosary, M., & Alnajay, H. (2013). Women׳s awareness and attitude toward epidural analgesia. J Biol Agric Healthc, 3(6).*

**Value of the data**

• The data provided by this study is of value and use to enhance women׳s knowledge and attitudes towards EA, antenatal clinics should offer more intensive information to women, including pamphlets and brochures that would provide appropriate and culturally oriented information about the current issue.
• The data shows that an efficient educational program through the use of media can be of important to implement.
• An intervention study would be necessary to evaluate the impact of implementing the campaign on the women׳s knowledge and attitudes towards EA,
• Findings were coherent with former studies, therefor making the data in this study of interest to other researchers.

## 1 Data

The data available in this study include demographic data, knowledge and attitudes questionnaires towards EA.

Analysis of the socio-demographic data of the study sample revealed that 44.5% of women aged 18 to 24 years old, 32.5% aged 25 to 34 years old and 23% aged 35 years and older. The mean age was 32.4 years (SD 5.4). Most of the women were employed. With respect to academic achievement, 27% had finished high school and 47% had a university bachelor degree. As for gravidity, 74.5% of the women participating in this research were multigravida, and 25.5% were primigravida. Concerning the income, 60% had a monthly income between 800,000 and 1,200,000 Lebanese Pounds and 32.5% had monthly earnings > 1,200,000 Lebanese Pounds. Demographic characteristics of the sample are shown in Table 1.

**Table 1:** Demographic characteristics of the sample.

**Item**	**Number (%) N=200**
Age	
18–24	91 (30.5)
25–34	48 (45.5)
35–45	61 (24)
Education	
Illiterate	13 (6.5)
Intermediate	7 (3.5)
High school	54 (27)
Bachelor	94 (47)
Post Bachelor education	32 (16)
Occupation	
Employed	140 (70)
Unemployed	60 (30)
Parity	
Multigravida	149 (74.5)
Primigravida	51 (25.5)
Income	
Below 800,000 LBP	15 (7.5)
800,000–1,200,000 LBP	120 (60)
Above1,200,000 LBP	65 (32.5)
Past labor with EA	
Yes	72 (38.1)
No	117 (61.9)

Research question 1: What is the women׳s level of knowledge concerning EA?

Women displayed an adequate extent of knowledge on the majority of the EA knowledge scale elements. High scores were related to: - Definition of EA (90%), – Who is supposed to administer EA (95.9%), – EA is the most prevalent and most efficient technique of labor pain management (70.2%), – The EA insertion does not induce more pain than the process of labor itself (70%), – EA decreases birth pain and enables the pregnant women to push as necessary (72%), – Participant must accept and sign a consent form for receiving EA during delivery (72%), – EA does not pose risks for the baby (54.5%), – EA should be accessible for women in labor (90%), and – EA can originate muscle weakness in the legs of the mother (52%). In contrast, there was a frustrating lack of knowledge with respect to the remaining elements of the EA knowledge scale. Table 2 represents knowledge of EA among women.

**Table 2:** Knowledge of EA.

**Items**	**Number (%)**			
	**Yes**	**No**		**Not sure**
EA is an injection of a local anesthesia through a catheter into the epidural space of the spine	180 (90%)		17 (8.5%)	3 (1.5%)
Any physician or nurse can administer the EA	1 (0.5%)		189 (95.9%)	7 (3.6%)
Contractions become weak or stop completely after administration of EA	84 (42%)		61 (30.5%)	55 (27.5%)
EA is the most frequently used and most effective way of relieving labor pain	139 (70.2%)		23 (11.6%)	36 (18.2%)
EA increase the risk for having C section	22 (11%)		105 (52.5%)	73 (36.5%)
The EA insertion in more painful than the labor pain itself	17 (8.5%)		140 (70%)	43 (21.5%)
EA reduce labor pain and allow the mother to bush when needed	144 (72%)		14 (7%)	42 (21%)
Women should agree and provide a consent for having EA at labor	144 (72%)		27 (13.5%)	29 (14.5%)
EA is risky for the baby	13 (6.5%)		109 (54.5%)	78 (39%)
EA can cause headache, fever, and lower blood pressure of the mother	78 (39%)		50 (25%)	72 (36%)
EA can cause muscle weakness in the lower limbs of the mother	104 (52%)		50 (25%)	46(23%)
EA should be an available option for women at delivery	180 (90%)		9 (4.5%)	11(5.5%)

Research question 2: From what sources of information did the women acquire their knowledge concerning EA?

The analysis revealed that 56% of information sources came from obstetricians, 11% from anesthesiologists, 17% from media and reading and 7% from antenatal clinics.

Research question 3: What are women׳s perspectives regarding the use of EA in prospective deliveries?

The majority of women stated that they are ‘‘not sure” about using EA in prospective deliveries (52.5%), while 31% of women ‘‘Agreed” and 16.5% ‘‘Disagreed”. Women who responded by disagreeing to the use of EA in subsequent births were inquired about the reasons behind their decision, and they answered that they were either guided by relatives and friends to avoid it or they wished to endure natural birth pain. Figure 1 shows attitudes of women toward EA.

Research question 4: Do demographic elements and knowledge affect the women׳s attitudes towards EA?

The factors that were significantly affecting the attitudes towards EA were the levels of education (χ2: 33.321, P<0.000), income (χ2: 26.969, P<0.000), parity (χ2: 35.032, P<0.000), the source of information for EA (χ2: 30.825, P: 0.001), previous experience with EA (χ2: 8.243, P: 0.016). More specifically, higher education, higher income, 2 or 3 previous labors, receiving information from obstetricians and having a previous experience with EA were linked with positive attitudes towards EA.

Concerning the dependency of the 12 knowledge items on attitudes towards EA, the items that showed significance were: knowledge of what EA is (χ2: 10.473, P: 0.033), the idea that contractions can stop because of EA (χ2: 23.109, P<0.000), the idea that EA can increase risk for cesarean section (χ2: 10.313, P: 0.035), the fact that EA is not causing more pain that labor itself (χ2: 10.275, P: 0.036), the fact that EA decreases delivery pain and enables mother to push (χ2: 11.889, P: 0.018), and the fact that EA is not risky for the baby (χ2: 26.100, P<0.000).

The logistic regression confirmed the significance of the previously mentioned factors as predictors of positive attitudes towards EA: higher education, higher income and 2–3 previous labors.

## 2 Experimental design, materials, and methods

### 2.1 Design

A quantitative approach using descriptive correlational cross-sectional design was adopted for this study to answer 4 research questions (1) What is the women׳s level of knowledge concerning EA, (2) From what sources of information did the women acquire their knowledge concerning EA (3) What are women׳s perspectives regarding the use of EA in prospective deliveries, and (4) Do demographic elements and knowledge affect the women׳s attitudes towards EA.

### 2.2 Sample and settings

A sample of 200 women in childbearing age (22–45 years old) who either have or do not have children participated in this study. Women were questioned by the researchers in person after accepting to take part in the study. Questionnaires were completed with women at shopping centers, cafeterias, styling salons and outpatient clinics in Lebanon. Women were requested to sign the consent form that integrated information related to the title of the study, its purpose and its significance.

### 2.3 Questionnaires

The research questionnaire incorporated socio-demographic data of the women such as age, academic level, job, parity, and medical data concerning previous surgical experience using EA. Knowledge of EA questionnaire was developed by the researchers based on prior studies with comparable aims [1], [2], [3]. The questionnaire comprises 12 elements on 3-points with (0) not sure, (1) no, and (2) yes. The scores vary from 1 to 24, the better the score, the better the women׳s knowledge regarding EA. The 12 elements are: (1) EA is an injection of a local anesthesia through a catheter into the epidural space of the spine, (2) Any physician or nurse can administer the EA, (3) Uterine contractions become subtler or cease fully after administering EA, (4) EA is the most prevalent and most efficient technique of labor pain management, (5) EA heightens the potential for having a cesarean section, (6) The EA insertion induces more pain than the process of labor itself, (7) EA decreases birth pain and enables the mother to push when necessary, (8) Women should accept and sign a consent form for receiving EA during delivery, (9) EA poses risks for the baby, (10) EA can cause headache, fever and lower blood pressure of the mother, (11) EA can cause muscle weakness in the lower limb of the mother, (12) and EA should be accessible for women in labor. Women were questioned about origins of their information about EA. Answers for sources of women׳s knowledge were classified as: media and/or reading, from family members/relatives and friends, from antenatal clinics, and from past experience. Attitudes toward EA were measured by using a sole question: “Would you use EA in any subsequent deliveries?” Answers for this question were (1) yes, (2) no, and (3) not sure. If a participant responded by “no” for this inquiry, she was required to provide explanation by writing the reason. An anesthesiologist, a PhD holder from nursing faculty and a nurse specialist in pain management have revised the research questionnaires. A pilot study was carried out on 20 participants who weren’t included in the main study prior to the data gathering and changes were made.

### 2.4 Statistical analysis

Categorical variables were expressed as number and percentage. Dependency between categorical variables was tested using the chi-square test. Logistic regression was used to identify the predictors of the attitudes towards EA in a subsequent delivery (dependent variable). SPSS version 20 and the R software were used for the analyses. The threshold for significance was set at 0.05.
