# Supplementary material for: Lebanese women׳s awareness and attitude toward epidural anesthesia during labor
Source: Data Brief. 2018 May 23;19:530–4. doi: 10.1016/j.dib.2018.05.051 (PMC5997907; doi:10.1016/j.dib.2018.05.051)
Supplement: Supplementary file 1 — Supporting information [file mmc1.docx]

**Conflict of Interest Statement**

**Title: Lebanese women’s’ awareness and attitude toward epidural analgesia during labor**

The authors whose names are listed immediately below certify that they have NO affiliations with or involvement in any organization or entity with any financial interest (such as honoraria; educational grants; participation in speakers’ bureaus; membership, employment, consultancies, stock ownership, or other equity interest; and expert testimony or patent-licensing arrangements), or non-financial interest (such as personal or professional relationships, affiliations, knowledge or beliefs) in the subject matter or materials discussed in this manuscript.

[Mirna A. Fawaz](http://www.scirp.org/journal/articles.aspx?searchCode=Mirna+A.++Fawaz&searchField=authors&page=1)^1^, Salah Malas^2^

1 Beirut Arab University, Faculty of Health Sciences, Lebanon, mirnafawaz@bau.edu.lb

2 Beirut Arab University, Faculty of Medicine, Department of Obstetrics and Gynecology, Lebanon, [s.malas@bau.edu.lb](mailto:s.malas@bau.edu.lb)
